# Bone health effects of androgen-deprivation therapy and androgen receptor inhibitors in patients with nonmetastatic castration-resistant prostate cancer

**DOI:** 10.1038/s41391-020-00296-y

**Published:** 2020-10-07

**Authors:** Arif Hussain, Abhishek Tripathi, Christopher Pieczonka, Diane Cope, Andrea McNatty, Christopher Logothetis, Theresa Guise

**Affiliations:** 1 University of Maryland Greenebaum Cancer Center and Baltimore VA Medical System, Baltimore, MD USA; 2 University of Oklahoma Health Sciences Center, Oklahoma City, OK USA; 3 AMP Urology, Syracuse, NY USA; 4 Florida Cancer Specialists and Research Institute, Fort Myers, FL USA; 5 Mayo Clinic AZ, Scottsdale, AZ USA; 6 MD Anderson Cancer Center, Houston, TX USA; 7 Indiana University School of Medicine, Indianapolis, IN USA

**Keywords:** Prostate cancer, Outcomes research

## Abstract

**Background:**

Osteoporosis is a skeletal disorder characterized by compromised bone strength, resulting in increased fracture risk. Patients with prostate cancer may have multiple risk factors contributing to bone fragility: advanced age, hypogonadism, and long-term use of androgen-deprivation therapy. Despite absence of metastatic disease, patients with nonmetastatic castrate-resistant prostate cancer receiving newer androgen receptor inhibitors can experience decreased bone mineral density. A systematic approach to bone health care has been hampered by a simplistic view that does not account for heterogeneity among prostate cancer patients or treatments they receive. This review aims to raise awareness in oncology and urology communities regarding the complexity of bone health, and to provide a framework for management strategies for patients with nonmetastatic castrate-resistant prostate cancer receiving androgen receptor inhibitor treatment.

**Methods:**

We searched peer-reviewed literature on the PubMed database using key words “androgen-deprivation therapy,” “androgen receptor inhibitors,” “bone,” “bone complications,” and “nonmetastatic prostate cancer” from 2000 to present.

**Results:**

We discuss how androgen inhibition affects bone health in patients with nonmetastatic castrate-resistant prostate cancer. We present data from phase 3 trials on the three approved androgen receptor inhibitors with regard to effects on bone. Finally, we present management strategies for maintenance of bone health.

**Conclusions:**

In patients with nonmetastatic castrate-resistant prostate cancer, aging, and antiandrogen therapy contribute to bone fragility. Newer androgen receptor inhibitors were associated with falls or fractures in a small subset of patients. Management guidelines include regular assessment of bone density, nutritional guidance, and use of antiresorptive bone health agents when warranted.

## Introduction

Osteoporosis is a skeletal disorder that compromises bone strength and increases the risk of fractures [1]. Broadly, in patients with prostate cancer, the interplay of multiple risk factors contributes to bone fragility. Of particular interest, risk factors associated with fragility fracture include hypogonadism; low body weight; current smoking; alcohol intake; vitamin D deficiency; low calcium intake; and long-term use of certain medications, such as glucocorticoids, anticoagulants, anticonvulsants, aromatase inhibitors, cancer chemotherapeutic drugs, and gonadotropin-releasing hormone agonists or antagonists [2]. Compared with a 13% risk for healthy men >50 years old, patients with prostate cancer have a 21–37% increased risk of fracture [3]. This increased risk may be accounted for by the compounding risk factors contributing to bone fragility and those associated with loss of bone mineral density (BMD), such as noted above [4]. Widespread use of androgen-deprivation therapy (ADT) contributes to a high prevalence of osteoporosis in up to 53% of men with prostate cancer [5]. Bone fragility in patients with metastases has been studied extensively; these studies have led to two US Food and Drug Administration (FDA) approvals, for zoledronic acid and denosumab, for the prevention of metastasis-related skeletal-related events (SREs) in this particularly high-risk population [4, 6, 7]. Distinct from metastasis-related SREs, fragility fractures can occur in patients independent of prostate cancer therapy, and thereby decrease patient quality of life [8].

Bone fragility and associated complications can also occur in the absence of documented metastases. Patients with nonmetastatic prostate cancer (nmPC) who receive continuous ADT have been reported to experience significant cumulative loss of BMD: 3.3 ± 0.7% in the lumbar spine, 2.1 ± 0.6% in the trochanter, and 1.8 ± 0.4% in the hip over 12 weeks [9]. BMD loss of 1.4–4.6% in the lumbar spine, 0.6–3.3% in the total hip, and 0.7–3.9% in the femoral neck annually among such patients has also been reported [10]. This translates into fractures as shown in a study of bone complications in 179,744 patients with prostate cancer (metastatic and nonmetastatic) who received ADT [11]. The study revealed that the hazard ratio (HR) for any fracture was 1.4 (95% CI 1.28–1.53), for hip fracture 1.38 (0.20–1.58), and for nonskeletal injury 1.01 (0.90–1.13). For patients not on ADT, these values were 0.97 (0.90–1.05), 0.95 (0.84–1.07), and 0.84 (0.77–0.92), respectively [11]. The Prostate Cancer Outcomes Study (PCOS) further explored the relationship between ADT duration and bone complications in a cohort of 3533 patients with nmPC who completed 15-year follow-up surveys to report development of fracture and use of bone-related medications [12]. Compared with untreated patients, those who received prolonged ADT (>1 year) had a higher odds of fracture (odds ratio [OR] 2.5, 95% CI 1.1–5.7), BMD testing (OR 5.9, 95% CI 3.0–12), and bone medication use (OR 4.3, 95% CI 2.3–8.0). The findings of the PCOS reflect the proportion of patients with nmCRPC (nonmetastatic castrate-resistant prostate cancer) who suffered bone complications during prolonged treatment with ADT [12].

In another study, patients with prostate cancer treated with ADT had an increased risk of any fracture (OR 2.83 [95% CI 2.52–3.17]) and of hip fracture requiring hospitalization (OR 1.82 [95% CI 1.44–2.30]) [13]. Excluding pathological fractures and spinal cord compression reduced the calculated overall OR to 1.47, still an elevated risk from ADT use [13]. Among the bone markers studied that are associated with increased osteoclastic bone destruction, N-telopeptide has been shown to be the most significant predictor of death in patients with prostate cancer (relative risk [RR] 3.25; 95% CI 2.26–4.68) and is further evidence that more bone destruction increases the risk of death [14]. Close monitoring of bone health for patients on ADT has been recommended, as fractures increase morbidity and risk of mortality when they occur [13].

Novel and more potent androgen receptor inhibitors (ARIs) are increasingly used in earlier disease settings, resulting in longer exposure times that, in turn, can result in increased risk of falls and fractures. The degree of increase risk of falls and fractures is different for the novel ARIs [15–17]. Collectively, these observations raise the concern that the increasingly prevalent use of second-generation antiandrogens in earlier disease states may increase the number of patients at risk for fracture. In older adults, increased fracture incidence also plays a role in decreased quality of life [8].

Identification and management of fragility fracture risk prior to actual fracture is important to the care of patients with prostate cancer. A systematic approach to bone health care for patients with prostate cancer has been hampered by a lack of standardized guidelines, lack of BMD testing, and gaps in education about: (a) adverse events (AEs) associated with ADTs, (b) diet and lifestyle on bone health, and (c) pharmacologic intervention to reduce fracture risk [4]. The purpose of this review is to raise awareness in the oncology and urology communities about the importance of bone health among patients with prostate cancer eligible for treatment with ARIs, to discuss the determinants of risk, and particularly to highlight emerging data about the management of fracture risk in patients. The review was prompted by the potential increased risk of bone complications in patients benefiting from newly approved life prolonging therapies that also increase bone fragility. The goal of the review is to provide a blueprint for developing multidisciplinary management strategies for improving bone health for patients with nmCRPC.

## Pathophysiology of bone fragility and changes due to aging and prostate cancer

**Fig. 1:**
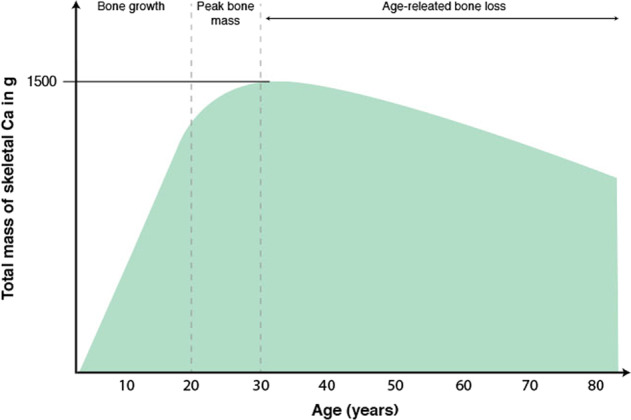
Adapted from [19] and [20] to show age-related bone loss in men. Santos et al. [19] is an open access article distributed under the terms of the Creative Commons CC BY license, which permits unrestricted use, distribution, and reproduction in any medium.

In patients with prostate cancer, three main drivers of fragility fracture risk are: (1) the aging process, (2) cancer treatment, primarily with ADT, and (3) cancer phenotype. Normally, peak BMD is achieved in young adulthood, generally around 20 years of age; it is maintained until ~40 years of age, and then declines for the reminder of one’s lifespan (Fig. 1) [18–20]. Fracture risk rises substantially with age to a degree that outpaces the effects attributed to loss of BMD [18]. Cancer phenotype refers to the variation between individuals with the same cancer diagnosis.

Several pathways are involved in age-related bone loss among men within the general population. A major contributor to BMD loss with aging is testosterone deficiency due to increases in sex hormone-binding globulin, which reduces free testosterone [18]. Testosterone has several effects on bone cells (osteoblasts, osteoclasts, and osteocytes), all of which express androgen receptors. Ligand binding of the androgen receptor stimulates osteoblast proliferation and suppresses apoptosis [21]. Androgen receptor activation by dihydrotestosterone of osteoclasts inhibits bone resorption in in vitro studies [21]. Decreased testosterone also leads to increased osteoblast-secreted RANKL (receptor activator of nuclear factor kappa-B ligand) levels, which then stimulates osteoclast-mediated bone resorption [21]. Another pathway to bone loss is the accumulation of impaired osteoblasts due to age-related telomere shortening, accumulation of oxidative stress, and DNA damage, all of which can lead to inefficient bone formation and remineralization [18].

In patients with prostate cancer, including nmCRPC, certain androgen-inhibiting treatments can contribute to bone fragility by causing accelerated osteoclastic bone destruction [4, 8]. Although rare, tumor-induced osteomalacia (TIO) may be involved as well. TIO is associated with increased production of fibroblast growth factor (FGF)-23, which results in impaired bone mineralization by inappropriate renal phosphate wasting, reduced calcium absorption and marked softening of bones [22]. Osteomalacia in general can be caused by insufficient calcium, phosphate or vitamin D and is associated with reduced bone density, but can be distinguished from osteoporosis by bone biopsy. Chronic vitamin D deficiency can also contribute to overall skeletal morbidity [22].

FGF signaling in general provides a positive feedback loop between bone cells and the tumor microenvironment of prostate cancer cells. In particular, blockade of the FGF-1 receptor in osteoblasts can partially mediate the antitumor effect of the vascular-endothelial growth factor inhibitor, dovitinib, a drug that also inhibits FGF-1 and FGF3 signaling [23]. Moreover, FGF-23 is a downstream target of FGF-1 signaling in osteocytes; therefore, blocking FGF-1 blocks FGF-23 [23]. A proof-of-principle study in patients with metastatic castration-resistant prostate cancer (mCRPC) showed that treatment with dovitinib reduced bone metastases: 26% of patients showed improvement on bone scan, with a median treatment duration of 19.9 weeks [23].

Additional considerations that contribute to bone fragility are the effects of androgen-deprivation and aging that decrease muscle mass and function, increase fat, and reduce mobility [24, 25]. Testosterone plays a key role in the preservation of muscle mass [24], whereas ADT contributes to muscle dysfunction and loss of muscle mass. In a study of 32 evaluable patients treated with gonadotropic releasing hormone agonists over 48 weeks, lean body mass decreased by 2.7% (*P* < 0.001); at the same time, body mass index increased 2.4% (*P* = 0.005) [26]. Untreated age-related muscle wasting may contribute to falls and fractures. Among persons ≥65 years, 5–13% have loss of muscle mass, and this proportion increases to 50% for those >80 years of age [25]. Mobility is also integral to overall bone health; acute immobilization has been shown to accelerate bone turnover and bone loss which, in turn, contribute to falls and fractures in the elderly. These interdependent effects on fracture risk: bone fragility, muscle weakness, and increased fall risk emphasize the need to carefully select therapeutic interventions in the context of underlying bone health. It should be noted that many of the above pathophysiologic processes associated with bone fragility are likely to be operative to varying degrees in patients with either nmCRPC or mCRPC.

## ARIs in nmCRPC and effects on bone: insights from clinical trial data

The approved next generation ARIs—apalutamide, darolutamide, and enzalutamide—have demonstrated efficacy in patients with nmCRPC in the SPARTAN, ARAMIS, and PROSPER phase 3 trials respectively; each trial also evaluated potential risk of falls and fractures [15–17]. These three agents were each added to ADT and evaluated against placebo in similarly designed studies of nmCRPC with prostate-specific antigen doubling time (PSADT) ≤10 months, with the primary endpoint being metastasis-free survival. Patients in these three trials were stratified by use of bone-sparing agents (not specified) at baseline, along with other criteria. Each trial favored addition of ARI to ADT in terms of statistically significant improvement in metastasis-free survival [15–17]. Updated data from the ARI trials also showed increase in overall survival compared with placebo (HR for apalutamide 0.78 [95% CI 0.64–0.96; *P* = 0.0161]) [27]; HR for darolutamide 0.69 [95% CI 0.53–0.88; *P* < 0.003] [28]; HR for enzalutamide 0.73 [95% CI 0.61–0.89; *P* = 0.001] [29].

Although comparisons across clinical trials are imprecise, the similar designs and placebo-control arms allow for some comparison of these agents to be made. SPARTAN (*N* = 1207) evaluated the efficacy of apalutamide in men with nmCRPC who had PSADT of ≤10 months [15]. In SPARTAN, 1207 enrolled patients were randomized to receive apalutamide or placebo. Falls and fractures occurred in 125 (15.6%) and 94 (11.7%) patients in the apalutamide arm, compared with 36 (9.0%) and 26 (6.5%) patients in the placebo arm, respectively (Table 1) [15]. A secondary analysis of SPARTAN data showed that patients ≥75 years of age had significantly higher risk of fractures and falls (HR 1.8, 95% CI 1.3–2.7), compared with patients <75 years of age [30].

**Table 1:** Falls, fractures, and other bone-related AEs in phase 3 trials for ARIs.

		Treatment arm			Placebo arm		
Study	Drug	Falls^a^ *n* (%)	Fractures *n* (%)	Other^b^ *n* (%)	Falls^a^ *n* (%)	Fractures *n* (%)	Other^b^ *n* (%)
SPARTAN (*N* = 1207) [15]	Apalutamide	125 (15.6)	94 (11.7)	NR	36 (9.0)	26 (6.5)	NR
ARAMIS (*N* = 1509) [17]	Darolutamide	40 (4.2)	40 (4.2)	139 (14.6)	26 (4.7)	20 (3.6)	68 (12.2)
PROSPER (*N* = 1401) [16, 28]	Enzalutamide	106 (11)	91 (10)	73 (8)	19 (4)	23 (5)	33 (7)

*AE* adverse event, *ARI* androgen receptor inhibitor, *NR* not reported.

^a^In SPARTAN, falls were deemed treatment-related by the investigators. In ARAMIS, falls included events recorded as accidents, and were determined to have been accidental falls.

^b^Other includes back pain in PROSPER, and back pain or pain in an extremity in ARAMIS.

In ARAMIS (*N* = 1509), which evaluated the efficacy and safety of darolutamide in men with nmCRPC and a PSADT of ≤10 months against placebo, AEs that occurred in the darolutamide arm included back pain (84 patients, 8.8%), pain in an extremity (55 patients, 5.8%), fractures (40 patients, 4.2%) and falls (40 patients, 4.2%) (Table 1) [17]. For patients who received placebo, back pain occurred in 50 (9.0%) patients, pain in an extremity occurred in 18 (3.2%), fractures in 20 (3.6%). patients and falls in 26 (4.7%) patients [17]. Although no secondary analysis or additional trial data for darolutamide are yet available, these data suggest that darolutamide may not significantly increase fracture incidence beyond any background risk.

The PROSPER trial (*N* = 1401) compared enzalutamide to placebo in men with nmCRPC and a PSADT of ≤10 months [16]. Among the AEs occurring in ≥5% of patients in the enzalutamide arm, falls and back pain occurred in 106 (11%) and 73 (8%) patients, respectively (Table 1) [16]. For patients who received placebo, falls and back pain occurred in 4% and 7%, respectively [16]. More fractures were reported for patients who received enzalutamide (10%) than placebo (5%) [31].

Another next generation hormonal agent, abiraterone, which primarily inhibits androgen biosynthesis but also has some ARI activity, has been evaluated in the smaller IMAAGEN phase 2 trial in patients with high-risk nmCRPC; it demonstrated a significant (87%) reduction in PSA level. At 48 months, 62% of patients were estimated radiographically to be progression-free. However, data on falls and fracture risks were not reported [32].

Comparison across these three trials is problematic; nevertheless, the initial experience from the large ARI phase 3 trials suggest that patients given darolutamide are less susceptible to falls and similar to placebo. Additional real-world experience will help further clarify the role of these next generation antiandrogen receptor targeting agents in terms of relative fracture and fall risks.

## Management strategies

The latest guidelines addressing bone health-related risks among prostate cancer patients on ADT are not applied uniformly in practice [4, 6, 10]. Thus, there is an unmet need for the oncology and urology communities to address bone health management, including in patients with nmCRPC, a disease state in which newer androgen receptor targeting agents are being increasingly incorporated in addition to standard ADT as part of patients’ overall treatment. Management strategies are summarized in Table 2.

**Table 2:** Management strategies.

Modality	Component	Evidence or comments	Dosing	References
Screening	Schedule	17,017 patients over 3 years showed only a minority were screened for BMD; screening rate varied from 15 to 20%. Those screened were older and fractures, osteoporosis was more common among them.	N/A	[29]
	DEXA	DEXA scores are based on *T* scores, where a *T* score of ≥−1 SD of the accepted mean reference value indicates normal BMD; *T* scores <−1 and >−2.5 SD indicate osteopenia, and *T* scores ≤−2.5 SD indicate osteoporosis (severe osteoporosis if also in the presence of ≥1 fragility fractures). Insurance coverage may limit how often patients undergo screening. For patients ages ≥65 years, Medicare part B insurance may cover DEXA scans every 2 years (or more frequently when prescribed by the physician) if preliminary X-rays show bone loss, or if patients suffered a previous fracture associated with loss of BMD.	N/A	[10, 52]
Exercise	High-intensity resistance training	20 weeks of high-intensity resistance training helped maintain BMD in 10 patients with mCRPC on ADT.	N/A	[30]
	Impact and resistance training vs. stretching	51 patients with CRPC treated with ADT were assigned to either a year of impact and resistance training or stretching. The men assigned to impact and resistance training showed significantly improved preservation of BMD at the L4 vertebrae (−0.4% for impact and resistance training compared with −3.1% for stretching, *P* = 0.03). The more intense regimen was well tolerated and showed positive effects on spinal BMD.	N/A	[31]
	Sports	109 patients were randomized to play football for 1 h twice/week, and the same number received usual care. Improvement in hip BMD was seen among patients who participated, but not in spinal BMD (*P* = 0.037).	N/A	[32]
Pharmacotherapy	Denosumab	Denosumab at 60 mg given every 6 months was shown to improve lumbar BMD significantly after 2 years compared with placebo: (+5.6% denosumab vs. −1.0% placebo; treatment difference 6.7% [95% CI 6.2–7.1]; *P* < 0.001). Denosumab significantly delayed time to first bone metastasis (HR 0.84 [0.71–0.98]; *P* = 0.032). Note that no bone-targeting agent has been approved for prevention of a first bone metastasis in any clinical setting.	60 mg every 6 months by subcutaneous injection	[6, 7]
	Zoledronic acid	Results of a systematic review and meta-analysis spanning the years 1946–2017 showed that zoledronic acid was effective in improving bone health in patients with nmCRPC.	5 mg IV yearly absent renal dysfunction	[4]
	Alendronate	Results of a systematic review and meta-analysis of 4 databases spanning the years 1800–2009 [sic] showed that alendronate decreased risk for developing osteoporosis (risk ratio 0.44; 95% CI 0.29–0.67) or fracture (0.51; 95% CI 0.1–2.69) relative to placebo in patients on ADT.	In the studies surveyed: 70 mg once/week per os	[53]
	Pamidronate	Results of a systematic review and meta-analysis of 4 databases spanning the years 1800–2009 [sic] showed that pamidronate increased lumbar spine (8.65 ± 6.85), total hip (8.65 ± 6.85), and trochanter (3.25 ± 0.63) BMD in patients on ADT after 12 months.	Per Smith et al.: 60 mg every 12 weeks IV	[9, 53]
Nutritional supplements	Calcium	Supplementation strongly recommended, especially with ZA or denosumab. To ensure adequate calcium intake, 3 or 4 daily servings of calcium-rich food should be consumed; if the total calcium consumed is <1000 mg to 1300 mg per day, then calcium supplements (600 mg) are advised. The recommended calcium intake for men 51–70 years is 1000 mg/day and for men >71 years, it is 1200 mg/day. If supplements are taken, they should be divided over the course of 3 daily meals for best absorption.	600 mg advised to reach total recommended Ca intake	[6, 33]
	Vitamin D	Supplementation strongly recommended, especially with ZA or denosumab. Vitamin D levels should be assessed prior to commencing therapy; if levels are <20 ng/mL, supplementation with 3000–5000 IU vitamin D per day for ≥6–12 weeks, followed by 800 IU daily maintenance should be considered. Supplementation should be provided to keep levels between 30 ng/mL and upper limit of normal for respective assay.	3000 to 5000 IU vitamin D daily for ≥6–12 weeks, followed by 800 IU daily	[6, 33]
	Protein	≥1.2 g of high-quality protein per kg body weight daily	N/A	[38]
Lifestyle modifications	Caffeine	Limit to ≤400 mg/day	N/A	[44]
	Alcohol	Limit to ≤2 drinks/day	N/A	[44]

*ADT* androgen-deprivation therapy, *BMD* bone mineral density, *CI* confidence interval, *CRPC* castration-resistant prostate cancer, *DEXA* dual-energy X-ray absorptiometry, *HR* hazard ratio, *IU* international units, *IV* intravenous, *NA* not applicable, *nm* nonmetastatic, *RR* relative risk; *SD* standard deviation, *ZA* zoledronic acid.

## Screening

Bone density screening is essential for early detection of patients at risk for SREs, but is not performed consistently in men [33]. Results of a study by the US Department of Veterans Affairs suggest that routine screening is warranted among patients with prostate cancer (Table 2) [33]. Dual-energy X-ray absorptiometry (DEXA) is the most widely used method to measure BMD. The World Health Organization (WHO) and many national health agencies recommend that BMD be monitored in patients with prostate cancer (Table 2) [10]. As per WHO guidelines, baseline DEXA scans are recommended. Although computed tomography and other imaging techniques might be used to assess bone mineralization, the widespread application to clinical practice is limited for these modalities [10].

## Exercise

Exercise has benefits in the maintenance of bone health during aging. During weight-bearing exercise, mechanosensors, such as stretch-activated ion channels, within osteocytes can trigger cascades leading to new bone deposition [19].

The effect of exercise in patients with nmCRPC treated with ARIs has not yet been studied, but some conclusions about the efficacy of exercise on preserving bone health may be drawn from prior studies on patients with localized prostate cancer. In one small study, patients who underwent high-intensity resistance-training maintained BMD, although none showed an increase in BMD (Table 2) [34]. In another study, patients with prostate cancer treated with ADT were assigned to either a year of impact and resistance training or stretching. The more intense training regimen was tolerated well by patients receiving ADT and showed positive effects on spinal BMD (Table 2) [35]. Recently, investigators evaluated sports participation as effective exercise for preservation of BMD in individuals with prostate cancer, of whom a proportion were on ADT [36]. Among the patients on ADT, there was statistically significant improvement in hip BMD among patients who participated in sports (Table 2), but not in spinal BMD [36]. A prospective trial to determine the effect of exercise among patients with prostate cancer treated with ADT has recently been completed [37]. This was a year-long randomized controlled study comparing exercise or nutritional supplementation (calcium, vitamin D; *n* = 51) versus no intervention (usual care group; *n* = 51) [37]. Exercise consisted of aerobic exercise, resistance training, and weight-bearing exercise [37]. Assessments of BMD were made by DEXA scan and bone strength determinations by peripheral quantitative computed tomography [37]. The trial has been completed and, as of this writing, final data are awaited.

Safe movement and exercise programs may be developed under the guidance of physical therapists for patients were warranted. As well, some clinicians may also recommend home resistance training programs.

## Pharmacotherapy

Pharmacotherapy for maintaining or improving bone health includes bone resorption inhibitors, bisphosphonates, and denosumab. Although the ultimate target for bone-protecting/bone-sparing agents (BPA) is the osteoclast, they differ in their mechanism of action and pharmacokinetics, which have important clinical implications. Bisphosphonates (i.e., bone mineral analogs) become part of the bone mineral matrix, whereas denosumab (a monoclonal antibody) binds to a cytokine ligand circulating outside bone cells (Table 3) [38]. Bisphosphonates, such as zoledronic acid, inhibit farnesyl pyrophosphate synthase, ultimately leading to osteoclast apoptosis. Zoledronic acid and other bisphosphonates are deposited into mineralized bone matrix, and taken up by bone-resorbing osteoclasts, thereby contributing to their cellular selectivity. The activity of denosumab, in contrast, is directed against RANKL, a cytokine that promotes osteoclast recruitment, maturation, activation, and survival [38]. Clearance of bisphosphonates is through bone turnover and for denosumab through the reticuloendothelial system (Table 3) [38]. The relevance of these BPAs in decreasing fracture risk has been underscored by the recent European Organisation for Research and Treatment of Cancer (EORTC) 1333/PEACE III trial that evaluated enzalutamide plus radium-223 versus enzalutamide alone in patients with asymptomatic/minimally symptomatic mCRPC; use of BPAs 6 weeks prior to initiating radium-223 significantly reduced the fracture rate in patients [39]. Although such a formal evaluation of BPAs in nmCRPC patients has not yet been done, data from the PEACE III trial in patients with mCRPC highlight the relevance of BPAs in modifying fracture risk among patients at risk.

**Table 3:** Comparison of bisphosphonates and denosumab [34].

Characteristic	Bisphosphonates	Denosumab
Target	Farnesyl pyrophosphate synthase inhibitors (nitrogen-containing bisphosphonates, e.g., zoledronic acid)	MoAb to RANKL
Site of action	Taken up by bone matrix	Systematically on osteoclast formation (blood and extracellular fluid)
Structure	Small molecule	MoAb
Effect on osteoclasts	Inhibits resorption by osteoclasts; inhibits osteoclast survival by inducing osteoclast apoptosis	Inhibits differentiation of precursors into osteoclasts; inhibits osteoclast function and survival
How given	Oral (alendronate) or intravenously (zoledronic acid and pamidronate)	Subcutaneous
Clearance from body	Initially cleared by kidney, and long-term via bone remodeling	Cleared via RES (half-life ~26 days)
Contraindications	• Pregnancy • Severe renal impairment (see dosing guidelines for individual bisphosphonates) • Hypocalcemia • Hypersensitivity to any component	• Pregnancy • Hypocalcemia • Hypersensitivity to any active component or excipient

Adapted from [34] with permission.

*BMD* bone mineral density, *MoAb* monoclonal antibody, *RANKL* receptor activator of nuclear factor kappa-B ligand, *RES* reticuloendothelial system.

Importantly, among patients with nmCRPC, BPAs have also been shown to have value in maintaining bone health [4]. Studies comparing their efficacy in preserving BMD in patients with prostate cancer have shown that intravenous zoledronic acid preserved BMD better than did other bisphosphonates or denosumab. It is important to note that patients in these studies were treated with ADT, and these results may not hold for patients treated with ARIs [40]. Denosumab, available from the manufacturer in a 60-mg formulation given every 6 months, has been shown to increase bone mass in men with nmPC receiving ADT who are at high risk of fracture (Table 3) [40]. In the three ARI trials discussed above, at baseline Fizazi et al. reported 3% (darolutamide) vs. 6% (placebo) of patients used bone-sparing agents [17], Smith et al. reported 10.2% vs. 9.7% [15], Hussain et al. reported 11% vs. 10% [16].

In deciding whether or when to commence use of BPAs, practitioners might assess the FRAX-estimated risk of developing a fragility fracture using femoral BMD [8]. It should be noted that FRAX-derived fracture probability has yet to be validated in patients with nmCRPC, and a consensus intervention threshold has not been established [8].

Considering that patients with prostate cancer are likely to be treated with BPAs for longer duration than was investigated in clinical trials, effects of their long-term use should be taken into account when managing bone health [41]. Hypocalcemia has been reported to occur more frequently with denosumab than with zoledronic acid; it can be prevented with adequate calcium and vitamin D supplementation [41]. Recommended supplementation doses for when serum 25-hydroxyvitamin D2 and D3 levels are lower than 30 ng/mL are outlined in Table 2: if levels are <20 ng/mL, supplementation with 3000–5000 IU vitamin D per day for at least 6–12 weeks followed by 800 IU daily maintenance should be considered [6, 42]. Vitamin D levels should be monitored every 6 months during drug treatment. A second condition, osteonecrosis of the jaw (ONJ), occurs relatively infrequently among patients with prostate cancer treated with denosumab or zoledronic acid [43]. A composite analysis of patients with breast and prostate cancer treated with these agents showed the incidence of ONJ in the first year to be 1.1%, rising to 2.2% in the second year, 3.7% in the third year, and 4.6% each year thereafter [41]. Good oral hygiene reduces the incidence of ONJ; oral health should be addressed before commencing treatment with bone health agents, and preventive measures may be implemented by a dental health professional if necessary [44]. In particular, invasive dental procedures should be completed, and ill-fitting appliances should be addressed to reduce the incidence of ONJ [6]. For the many patients who present with underlying renal dysfunction, close monitoring for hypocalcemia is warranted. Caution should be taken when prescribing zoledronic acid in patients with reduced renal function; in these patients, doses should be adjusted accordingly. Denosumab does not need dose adjustments in patients with impaired renal function.

Finally, a treatment plan for duration and discontinuation of therapy should be developed. An important, but often not considered, aspect of denosumab treatment is the reported development of rebound osteoclastogenesis, which occurs when denosumab is discontinued [45]. Osteoclastogenesis can result in rapid bone loss and fracture in the year following cessation of denosumab therapy. Such increases in bone destruction can also enrich the local bone microenvironment to fuel cancer growth if tumor cells are present in the bone microenvironment. This poses a theoretical risk for bone metastases. This rapid bone loss does not occur when zoledronic acid is discontinued because the drug has a long half-life in bone. Thus, if denosumab is discontinued, zoledronic acid should be given, administered at a time when bone destruction recurs, to prevent the rapid bone loss. Currently, there are no clinical guidelines on how to cease denosumab treatment [46]. As such, health care providers should consider consultation with a specialist in metabolic bone disease to guide the transition of denosumab to zoledronic acid or another BPA [45, 46]. Currently available data suggest that administering zoledronic acid within 7–8 months or sooner after the last dose of denosumab may be best to minimize bone loss [47].

## Nutritional considerations and supplementation

Guidelines for nutritional supplementation have not yet been developed for nmCRPC treated with ARIs, as these drugs are too recent for specific studies to have been completed. Some insights may be gleaned from prior experience with older forms of androgen inhibition (ADT and antiandrogens), and the use of nutritional supplementation to help mitigate AEs on bone health in patients with prostate cancer. A systematic review of the literature showed that adequate nutrition during ADT may have helped mitigate treatment-related AEs on bone and that patients require guidance to help them follow a diet that will meet their complete nutritional needs [48].

A randomized phase 2 trial of patients with prostate cancer who received ADT examined educational strategies (family physician, bone health support care, or usual care) to assess compliance with methods directed toward improving bone health [49]. Secondary efficacy outcomes included vitamin D (800 mg to 2000 IU daily) and calcium (1000–1200 mg daily) use [48, 49]. Family physician and bone health support care educational strategies were found to improve bone health, including higher compliance for intake of calcium [49]. Extension of similar studies to ARIs might be a future consideration. Vitamin D should be recommended based on patient serum 25-hydroxyvitamin-D2 and -D3 levels, calcium intake should be at least 1000 mg/day (through food and/or supplements), and protein intake should be roughly 75 g/day [42]. Based on our collective clinical experience, several strategies are proposed to ensure adequate vitamin D, calcium, and protein intake, summarized in Table 2.

## Other lifestyle modifications

The positive effects of pharmacotherapy, exercise, and sufficient calcium and vitamin D intake may be augmented by additional changes to lifestyle. Most common recommendations include limiting caffeine consumption and imbibing only moderate amounts of alcohol [48]. Specific dietary recommendations have not been offered in the literature, although the importance of educating patients about the details outlined above (quantities of caffeine, alcohol, importance of regular exercise, and among others) should not be overlooked when discussing bone health with patients [48].

## Summary and conclusions

Older men, including those with prostate cancer, are at increased risk of diseases associated with decreased BMD and increased bone fragility and, consequently, are at increased fracture risk. Treatments that inhibit androgen production and androgen action have been associated with bone loss. For these reasons, the effects of ARI on bone health should be included when discussing treatment options for patients with nmCRPC. Bone health agents may be prescribed for patients at increased risk for fractures. In patients with nmCRPC, aging and androgen-inhibiting treatment both contribute to bone fragility. The increased risk of fractures following androgen-inhibition is due to increased osteoclastic bone destruction in addition to muscle dysfunction and increased risk of fall [4]. Reduced testosterone has a negative impact on muscle mass, which may lead to less mobility, further contributing to decreased BMD [24, 25, 50].

Clinical trial data for the approved ARIs apalutamide, darolutamide, and enzalutamide showed that falls and fractures occurred in a minority of patients with nmCRPC treated with these drugs; of the three, darolutamide was associated with the lowest incidence of falls and fractures (Table 1) [15, 16, 51]. However, this is not to say that one agent is associated with less fracture risk than the others, and as noted above updated data from these trials show that all three ARIs discussed are associated with increased overall survival compared with placebo, providing important treatment options for high-risk nmCRPC patients [27–29].

Management strategies for patients with nmCRPC treated with ARIs should include baseline and regular screening by DEXA [10], and lifestyle modifications, including recommendations for safe movement, and adequate and safe exercise [34, 35]. In addition, bone health agents may be prescribed, either denosumab or a bisphosphonate, both of which have been studied in patients with nmCRPC [4]. Nutritional assessment is important to ensure that patients have adequate intake of protein, calcium, and vitamin D. Caffeine and alcohol should be avoided. A multidisciplinary assessment and approach to management is likely to lead to optimal outcomes, including bone health among patients with nmCRPC.
